# Independence of Echo-Threshold and Echo-Delay in the Barn Owl

**DOI:** 10.1371/journal.pone.0003598

**Published:** 2008-10-31

**Authors:** Brian S. Nelson, Terry T. Takahashi

**Affiliations:** Institute of Neuroscience, University of Oregon, Eugene, Oregon, United States of America; Ludwig Maximilians University Munich, Germany

## Abstract

Despite their prevalence in nature, echoes are not perceived as events separate from the sounds arriving directly from an active source, until the echo's delay is long. We measured the head-saccades of barn owls and the responses of neurons in their auditory space-maps while presenting a long duration noise-burst and a simulated echo. Under this paradigm, there were two possible stimulus segments that could potentially signal the location of the echo. One was at the onset of the echo; the other, after the offset of the direct (leading) sound, when only the echo was present. By lengthening the echo's duration, independently of its delay, spikes and saccades were evoked by the source of the echo even at delays that normally evoked saccades to only the direct source. An echo's location thus appears to be signaled by the neural response evoked after the offset of the direct sound.

## Introduction

In nature, sounds of interest are often followed by reflections from nearby objects. Yet, sounds arriving directly from the actively-emitting source dominate spatial perception. This phenomenon, known as *localization dominance*, is a major component of the *precedence effect*, a collection of auditory phenomena thought to allow for the segregation of direct sound from reflections [1]–[4]. As the delay between a direct sound and a reflection increases, subjects begin to report having heard the reflection. Localization dominance thus ends and *echo-threshold* is said to have been reached [5]. What causes the reflection to become perceptually salient?

The precedence effect is typically studied using clicks, which helps to avoid the acoustical superposition of the leading and lagging sounds [although see 6], [7]. Physiological studies show that a cell's response to a lagging click is weak when the delay is short but that this response increases when the delay is long [8]–[16]. Echo-threshold is therefore thought to be related to the inter-click interval at which the strength of the response to the lagging click approaches that to the leading click.

**Figure 1:**
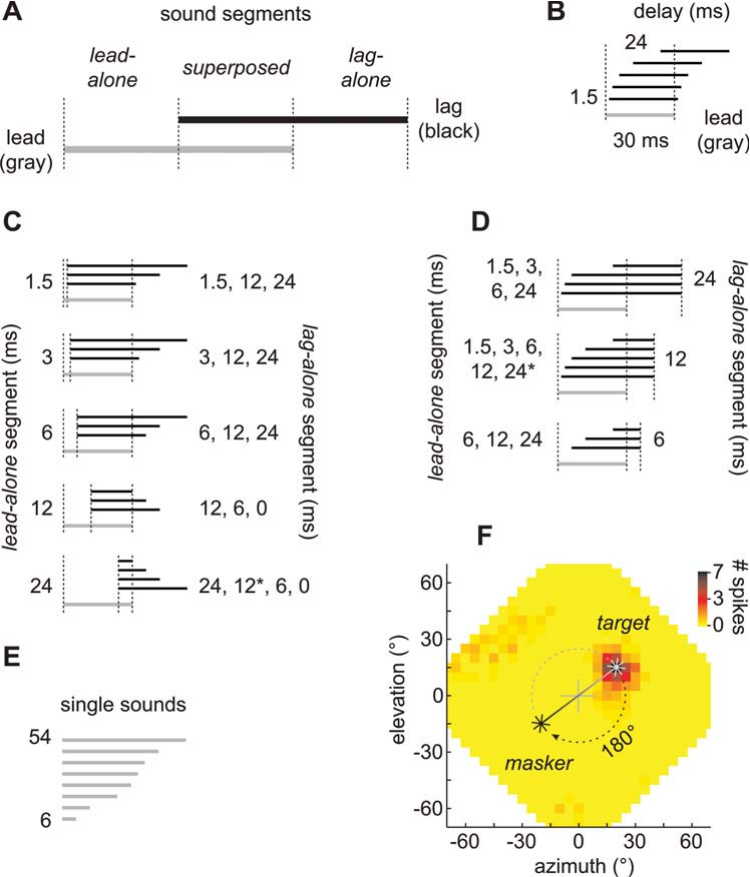
Stimulus configurations. (A) Overlapping lead (direct) and lag (simulated reflection) sounds. The temporal overlap defines periods of time during which both sounds were superposed, flanked by periods when the lead or lag sources were present alone. (B) Stimuli presented in the standard precedence effect, paradigm. The lead (gray) and lag (black) sounds were of equal length (30 ms) and onset-delay was 1.5, 3, 6, 12, or 24 ms. (C) Stimuli in which *lag-alone* segments were experimentally lengthened or shortened while maintaining a constant duration *lead-alone* segment (constant lead/lag delay). Lead and lag sounds were of unequal lengths. (D) Stimuli in which *lead-alone* segments were experimentally lengthened or shortened while maintaining a constant duration *lag-alone* segment (converse of C). When the *lead-alone* segment was 24 ms, the length of the *lag-alone* segment was shortened to 12 ms only in our physiological experiments (indicated by asterisks). (E) Single-source sounds among which paired-source stimuli were randomly interspersed in our behavioral experiments. Their durations were roved from 6–54 ms to invalidate duration as a possible cue. (F) Placement of sound sources in our physiological experiments. The plot represents the frontal hemisphere of the owl's auditory space [29]. Positive azimuths and elevations correspond to loci to the right and above an owl, respectively. A cell's SRF is shown in pseudo-color along with a scale bar indicating the average spike number over 4 repetitions. The source in the optimal location within the cell's SRF is referred to as the *target*. A second source placed at a location diametrically opposed across the owl's center of gaze from the target is referred to as the *masker*. In the experiments, the *target* or *masker* could lead, allowing us to examine a cell's response to simulated direct sounds and echoes.

Although clicks afford advantages for experimentation, sounds in nature will often overlap temporally with reflections that arrive after short delays. As a result, there is a period of time when both leading and lagging sounds are present, the *superposed* segment, flanked by the *lead-alone* and *lag-alone* segments (Fig. 1A). What determines the echo-threshold in this case? On the one hand, it could be determined by a neural response, evoked during the *superposed* segment, at the onset of the lagging sound. In this case, echo-threshold is expected to correlate well with the length of the *lead-alone* segment. Alternatively, echo-threshold could correspond to a neural response during the *lag-alone* segment [17]. In this case, echo-threshold is expected to correlate well with the length of the *lag-alone* segment.

We investigated these alternative hypotheses in the barn owl, Tyto alba, an auditory predator whose sound localization is guided by activity on a topographic representation of auditory space in the external nucleus of its inferior colliculus [ICx], [18,19]. Lesions of this auditory space map lead to scotoma-like defects in sound localization [20] and the resolution of its neurons can account for the owl's behavioral spatial acuity [21], [22].

Owls, like humans and other species [23]–[27], appear to experience localization dominance. They turn their heads toward the leading source for delays less than ∼10 ms and, as the delay increases, the proportion of saccades to the lag increases [12], [28]. Correspondingly, space map neurons respond weakly to lagging sources at delays less than ∼10 ms, but the responses increase for longer delays [12], [17].

We show that the responses of space-specific neurons evoked during the *lag-alone* segment, but not during the *superposed* segment, scale with delay. Suspecting that the *lag-alone* segment was too short to evoke a sufficient neural response when the delay was short, we experimentally lengthened this segment and found that the response increased regardless of the delay at the onset of the stimulus (onset-delay). Using stimuli manipulated thus, we found that the proportion of lag-directed saccades increased with the length of the *lag-alone* segment and regardless of the onset-delay.

## Results

### Physiology

Our conclusions are based on the recordings from 39 neurons isolated in the auditory space maps of 3 owls (owl 1029, N = 23; owl 1027, N = 12; owl 942, N = 4). All cells had well-circumscribed spatial receptive fields (SRF; Fig. 1F) when assessed with 100 ms noise-bursts presented in virtual auditory space (VAS) [18], [21], [29]. We refer to the stimulus that was presented from the center of each cell's SRF as the “*target*” and the stimulus that was presented from outside the SRF as the “*masker*” (Fig. 1F) [17]. As in our behavioral experiments (see below), the location of the masker was diametrically opposed to that of the target in polar coordinates (Fig. 1F). The leading sound's duration was always 30 ms. Onset-delays of 1.5, 3, 6, 12 or 24 ms were tested, resulting in *lag-alone*, *lead-alone* and *superposed* segments of variable lengths (Fig. 1B). In all cases, the noise stimuli were broadband (2–11 kHz). Except for when the stimuli were presented simultaneously (i.e., with no onset-delay), targets and maskers were correlated in the sense that they were produced using identical noise-bursts prior to the introduction of each delay.

**Figure 2:**
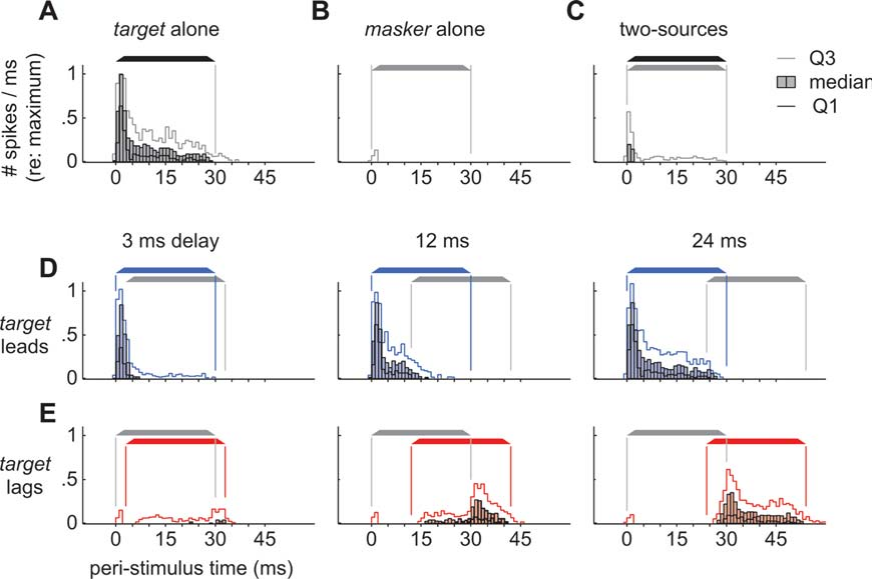
Peri-stimulus time histograms (PSTHs) showing neural responses evoked by a subset of stimuli. Each bar shows the median neural response (>50 repetitions/cell; 1-ms bins). Thin lines show the first and third quartiles (Q1, Q3). Each cell's response was normalized to the maximum number of spikes evoked, within a single bin (usually the first or second bin after 0-ms), by 30 ms noise-bursts presented from the center of each cell's SRF [11]. (A) Responses evoked by a single 30 ms *target*. (B) Responses evoked by a single 30 ms *masker*. (C) Responses evoked by two, simultaneous, uncorrelated, noise-bursts presented from both the *masker* and *target* loci. (D) Responses evoked when the *target* led by 3, 12 or 24 ms. (E) Responses evoked when the *target* lagged.

Histograms compiled from the responses of all neurons in our sample to representative targets and maskers are shown in Figure 2. The responses of a single representative neuron are shown in the Supplemental Materials (Figure S1). The height of each filled bar in Figure 2 shows the median normalized firing-rate within each 1-ms bin. Targets evoked strong responses (Fig. 2A), while maskers, presented alone, evoked weak responses (Fig. 2B; see also Fig. 1F). Because spontaneous firing-rates were low in all of our cells (spikes/ms/cell: median = 0.00028, first quartile = 0, third quartile = 0.0017), we cannot say whether or not the masker, by itself, had an inhibitory influence. Figure 2C depicts the neurons' responses to two, *simultaneous*, uncorrelated, noise-bursts presented from both the masker and target loci. Responses to these stimuli were weak due to the superposition of the two sources' waveforms, which, in turn, decorrelates the signals binaurally [30]–[33]. The second row of PSTHs (Fig. 2D) shows the median responses of sampled neurons to lead/lag pairs when targets led by 3, 12, or 24 ms. Overall spike counts increased with delay, but most of this increase was due to an increase in the length of time, from the start of the stimulus, during which only the leading target was present (the lead-alone segment or the onset-delay). The responses decreased as soon as the lagging masker was activated. The bottom row of PSTHs (Fig. 2E) shows the median responses when targets *lagged*. Responses increased as soon as the masker, which led, was deactivated and continued for a period of time that was determined by the length of the lag-alone segment (Fig. 2E).

**Figure 3:**
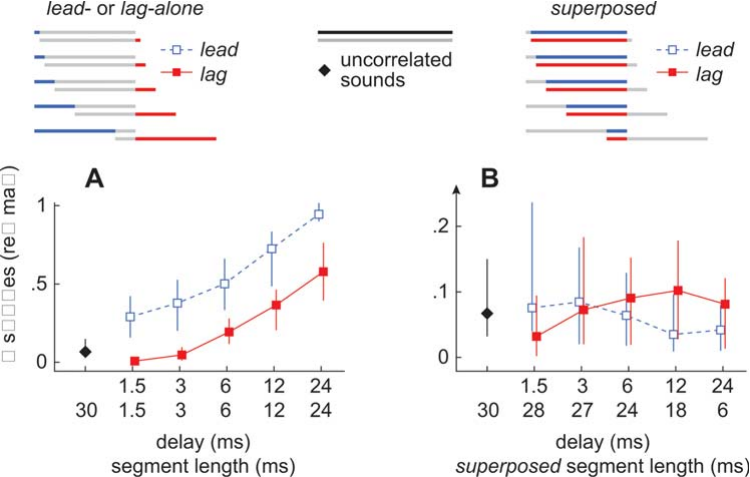
Summary of neural responses evoked during the *lead-alone*, *lag-alone*, and *superposed* segments. (A) Responses evoked during the *lead-alone* (open, blue, squares) and *lag-alone* (closed, red, squares) segments. Each data point represents the median number of spikes, normalized to the average response evoked, in each cell, by 30 ms sounds (>50 repetitions) presented from the center of its SRF [11]. Vertical lines indicate the first and third quartiles of each response. The upper row of numbers along the abscissa represents the onset-delay and the bottom row represents the length of each segment. (B) Responses evoked during the *superposed* segments when the *target* led (open, blue, squares) or lagged (closed, red, squares). Responses evoked by two, simultaneous, uncorrelated, noise-bursts from the *target* and *masker* loci are indicated by black diamonds (A,B). Note that the ordinate axis in panel B is expanded relative to that of panel A.

The recovery of neuronal responses to lagging sound sources has typically been attributed to the onset-delay [8]–[15], which, in our paradigm, is equal to the length of the lead-alone segment. The alternative hypothesis is that the lag-alone segment accounts for this recovery. Responses evoked during the lag-alone and superposed segments are plotted against delay in Figure 3 (red, filled, markers) to determine, quantitatively, which segment best accounts for the recovery to the lag source. Responses evoked during lag-alone segments increased significantly with delay (Fig. 3A; lead: P<1⋅10^−6^; lag: P<1⋅10^−6^; df = 4; Kruskal-Wallis). In contrast, responses evoked during superposed segments did not vary significantly with delay (Fig. 3B; lead: P = 0.053; lag: P = 0.16, df = 4; Kruskal-Wallis). Furthermore, they did not differ from the responses evoked by two, simultaneous, uncorrelated, noise-bursts (black diamond, Fig. 3B; lead: P = 0.068; lag: P = 0.24, df = 4; Kruskal-Wallis) suggesting that the decrease in firing rate during the superposed segment can be explained by binaural decorrelation, and that inhibition need not be invoked. Taken together, the observations above are inconsistent with the idea that the delay, *per se*, accounts for the neuronal recovery. Instead, the recovery is best attributed to the *lag-alone* segment.

Next, we tested whether an increased response could be evoked if the lag-alone segment were lengthened independently of the delay at the onset of the stimulus (i.e., independently of the duration of the lead-alone segment; Figs. 1C,D). Similarly, we tested whether a decreased response could be evoked if the lag-alone segment were shortened independently of the delay at the onset of the stimulus.

**Figure 4:**
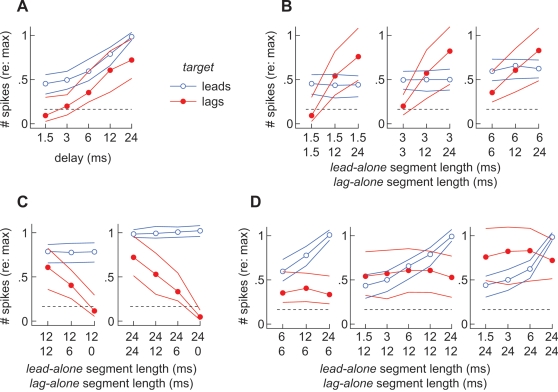
Summary of overall neural responses to lead and lag *targets*. (A) Responses evoked in the standard paradigm in which *lead-alone* and *lag-alone* segments were of equal length. (B) Responses evoked when *lag-alone* segments were experimentally lengthened. At each point along the abscissa, the number on top indicates the length of the *lead-alone* segment. The number underneath indicates the length of the *lag-alone* segment. (C) Responses evoked when *lag-alone* segments were experimentally shortened. (D) Responses shown here have been regrouped so that the length of the *lag-alone* segment is constant within each panel. All data points represent the median number of spikes evoked when the target led (blue lines) or lagged (red lines). Thin lines indicate the first and third quartiles of each response. Each value is normalized to the average response evoked, in each cell, by 30 ms sounds (>50 repetitions) presented from the center of its SRF [11]. The dashed horizontal line represents the median response evoked by two, simultaneous, uncorrelated, noise-bursts from *target* and *masker* loci.

Figure 4A shows the median responses evoked in our sample of 39 neurons in the standard paradigm where the lengths of the lead-alone and lag-alone segments were equal and when the target led (blue lines) or lagged (red lines; Fig. 1B). Leading targets evoked stronger responses than lagging targets and both responses increased with delay, as earlier studies have shown [8]–[15], [17].

Figure 4B shows conditions where the lag-alone segment was lengthened while the delay at each sound's onset was held constant (Fig. 1C). At each point along the abscissa, the number on top indicates the length of the lead-alone segment (onset-delay). The number underneath indicates the length of the lag-alone segment. In the left panel, the delay at the sound pair's onset was 1.5 ms, a value for which a lagging target evokes few if any spikes when the leading and lagging sounds are of equal length. As the lag-alone segment is independently lengthened to 12 and 24 ms, the firing rate increases (red lines). Similar results are shown for longer onset-delays of 3 and 6 ms. Meanwhile, the response to a leading target (blue lines) remains constant within each panel although there is variation from panel to panel, due to the increasing length of the lead-alone segment.

Figure 4C shows responses when the lag-alone segment was shortened (Fig. 1C). The delay at the onset of each stimulus was fixed at 12 ms (left) or 24 ms (right), values that normally elicited robust firing to the lag-alone segment. As the length of the lag-alone segment decreased, the response to the lag-alone segment diminished (red lines). The response evoked when the target led (blue lines) again remained constant within each panel.

The same responses are shown again in Figure 4D but the plots are regrouped so that the length of the lag-alone segment is constant within each panel (Fig. 1D). Thus plotted, it is apparent that the response to the lagging target (red lines) was independent of the length of the lead-alone segment and the superposed segment. Not surprisingly, the response to the leading target (blue lines) varied with the length of the lead-alone segment.

As a benchmark for echo-threshold, Yin [11] and others [9], [10] have reported the delay at which the lag's response increased to 50% of the response evoked, in each cell, by single sounds. Our experiments were not designed to derive this benchmark with precision. Given the delays tested, this “half-maximal” delay was between 12 and 24 ms when considering spikes evoked only during each stimulus' lag-alone segment (Fig. 3A) and was between 6 and 12 ms when we included spikes evoked, occasionally, while the stimuli were superposed (Fig. 4A).

### Behavior

Because the neural response to a lagging target was determined, almost entirely, by the length of its lag-alone segment, we predicted that this segment would determine the echo-threshold in our behavioral experiments. As is commonly done in human lateralization studies [e.g.], [ 34], [35]–[37], we determined the proportion of saccades toward the leading and lagging sources while manipulating the lag-alone segment independently of the delay (lead-alone segment).

### General properties of saccades

**Figure 5:**
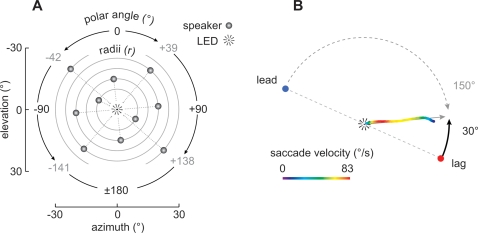
Behavioral paradigm. (A) Placement of 10 loudspeakers and a central fixation LED, in polar coordinates. In trials with a lead/lag pair, one of the pair of speakers was assigned a radius of 10°, 15°, 20°, 25°, or 30° and a random polar angle. The second member of the pair had an identical radius, but was 180° opposite the first speaker. Corresponding Cartesian coordinates (azimuth and elevation) are also shown. The stimulus paradigms used in the behavioral trials were identical to those shown in Fig. 1 for physiology. (B) Example of a head saccade. The saccade shown here was made with an unusually large error (30°) relative to that of the closest source, in this case, the lagging source. Despite this error, the angle of the saccade was far greater when compared with that of the leading source (150°), thereby allowing us to determine whether the saccade was lead- or lag-directed. The color scale indicates saccade velocity.

Our conclusions are based on observations in three owls, *C*, *S*, and *T* (N = 33–66 trials/condition/subject). The stimuli were the same as those in the physiology component (Figs. 1A–D), except that they were presented in the free field (Fig. 5A). Saccades to *single* sources had latencies and errors similar to those reported by Spitzer and Takahashi [28]. We also confirmed that latencies to paired sources were significantly longer than those to single sources and that saccade errors to lead and single sources were comparable for all lag-alone segments. Saccade errors and latencies are summarized in Supplemental Materials Figure S2. Unlike in this previous study, saccades to lag sources were comparable in error to those of leading and single sources for lag-alone segments of 12 and 24 ms. Errors in trials with shorter lag-alone segments (<3 ms) were larger, but because there were few lag-directed turns (due to localization dominance), this observation must be viewed with caution. This result was, nevertheless, consistent with another study quantifying spatial *discrimination* in the owl under simulated echoic conditions [33].

One difference between the present study and the earlier study of Spitzer and Takahashi [28] was that we did not observe “double saccades” in which the bird first turned toward one source (lag *or* lead) and then redirected its saccade toward the other. We attribute this to a difference in paradigms: In the present study, the speaker pairs were positioned symmetrically (Fig. 5A) about the center of the owl's gaze at the start of each trial. This speaker arrangement reduced amplitude differences between the two sounds arising from the birds' head-related transfer functions [29]. Since the speakers were positioned symmetrically (Fig. 5A), a double saccade would also entail a hairpin turn (∼180° reversal) that may be difficult to execute. In the previous study, the speaker pairs could be at any location relative to the bird's initial gaze, allowing for less drastic course-changes that were likely to be easier to execute. Our current paradigm was devised to eliminate these small course-changes because they are difficult to score in a lateralization-like paradigm.

### Saccades in paired-source trials

**Figure 6:**
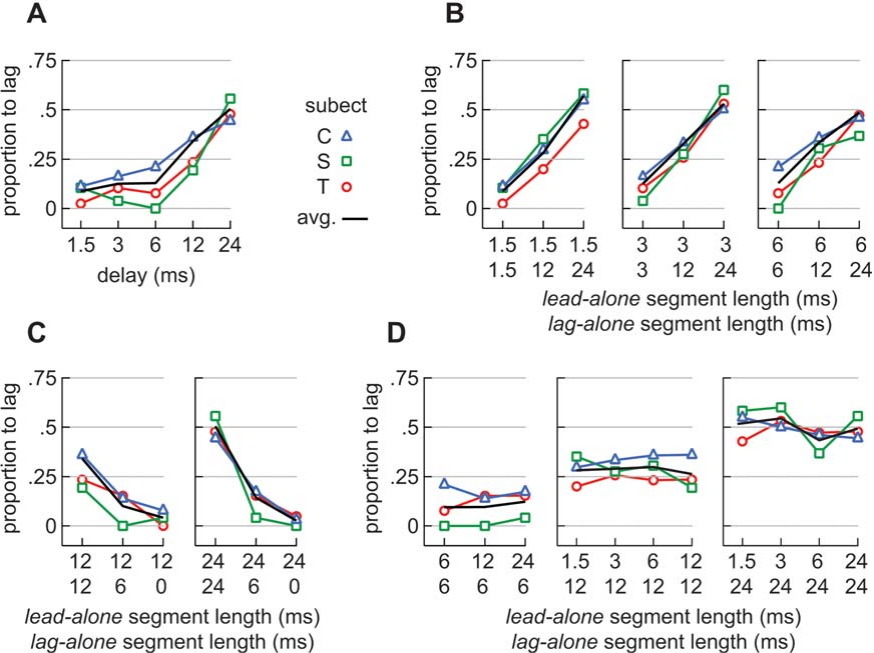
Proportions of trials on which saccades were lag-directed for three birds. (A) Proportion of lag-directed saccades in the standard paradigm plotted against the lead/lag delay. The black, dashed, line in this and other plots shows the average of all three subjects. (B) Proportion of lag-directed saccades observed when *lag-alone* segments were experimentally lengthened. At each point along the abscissa, the number on top indicates the length of the *lead-alone* segment (onset-delay). The number underneath indicates the length of the *lag-alone* segment. (C) Proportion of lag-directed saccades observed when *lag-alone* segments were experimentally shortened. (D) Proportions of lag-directed saccades shown here have been regrouped so that the length of the *lag-alone* segment is constant within each panel.

Results are shown in Figure 6. Figure 6A plots the proportion of saccades for each bird to the lagging source, against delay, in the standard paradigm where the lead and lag sounds were of equal lengths (Fig. 1B). Consistent with the results of an earlier study [28], the proportion of lag-directed turns remained low for delays up to 6 ms and increased for delays of 12 or 24 ms.

In studies designed to probe echo-threshold, human subjects are commonly asked to report the number of sources heard – a task for which the owls were not trained. We cannot, therefore, say whether the owls perceived both the lead and lag sounds on a given trial or just the one to which it turned. What is clear, however, is that for the longer delays, the proportion of lag-directed turns increased. Defined as the delay at which the lag source begins to influence the owl's behavior [9], [27], and given the delays tested, we can therefore say that the echo-threshold was between 6 and 12 ms, a range consistent with earlier studies [9], [12], [27], [28].

Figure 6B shows the proportion of lag-directed saccades when delay was held constant at 1.5, 3, and 6 ms and the length of the *lag-alone* segment was varied (Fig. 1C). At each point along the abscissa, the number on top indicates the length of the *lead-alone* segment (onset-delay). The number underneath indicates the length of the *lag-alone* segment. The data demonstrate that lengthening the *lag-alone* segment increased the frequency with which saccades were lag-directed (Fig. 6B; *subject C*: P = 0.02, *T*: P = 0.01, *S*: P = 0.008; contingency table analysis). Conversely, shortening the *lag-alone* segment decreased the frequency with which saccades were lag-directed (Fig. 6c; *subject C*: P = 0.002, *T*: P = 0.015, *S*: P = 0.02).

The data shown in Figures 6B,C are re-plotted in Figure 6D but are regrouped so that the length of the *lag-alone* segment is constant within each panel (Fig. 1D). The data show that the length of the *lead-alone* segment, and thus the onset-delay, did not influence the proportion of trials on which saccades were lag-directed (*subject C*: P = 0.6, *T*: P = 0.5, *S*: P = 0.2; contingency table analysis).

## Discussion

Our results suggest that in the owl, echo-threshold is related to the neural response on the space map elicited by the *lag-alone* segment. By lengthening this segment, we were able to evoke neural responses in the space map and saccades to the lagging source, even at short delays that evoked localization dominance in the standard experimental paradigm, i.e., when lead and lag sounds were equally long. We suggest that, in the standard paradigm, owls localize the lead source preferentially at short delays because the response to the lag-alone segment is weak. Echo-threshold is reached when the lag-alone segment, which would be equal in length to the onset-delay, is long enough to elicit significant activity on the space map. The idea that echo-threshold is determined by the onset-delay, *per se*, may thus be true only to the extent that this delay determines the length of the lag-alone segment under standard conditions.

The precedence effect is typically studied using clicks, and localization dominance is often attributed to a process by which the neurons responding to the leading click preempt the responses of neurons that represent other loci [8]–[17], [38]. Echo-threshold is reached, according to this view, when the lagging click arrives after this lateral inhibition-like process has subsided. Applying this idea to our stimuli, one might have expected echo-threshold to be related to the recovery of a neural response during the superposed segment, e.g., at the onset of the lagging sound. Our data are inconsistent with this hypothesis. First, the neural response during the *superposed* segment was independent of onset-delay. Had echo-threshold been related to the superposed segment, we should have seen an increased response, with delay, during the superposed segment (Fig. 3B). Instead, the neural response during the superposed segment could be explained, for all delays, by the presence of two uncorrelated noises and the resulting binaural decorrelation [30]–[33]. Second, we observed that the proportion of lag-directed saccades remained constant as long as the *lag-alone* segment's length was held constant and did not depend on the length of the *lead-alone* segment (Fig. 6D). Had the echo-threshold been related to the *lead-alone* or *superposed* segments, we should have seen an increase in lag-directed saccades as the delay increased while the length of the lag-alone segment was held constant.

Our results hint at the way in which activity on the space map might be read. One possible scenario is that the ratio with which saccades are lead- or lag-directed (*N_lag_/N_lead_*) is proportional to the ratio of the neural responses to the leading and lagging sources respectively (*R_lag_/R_lead_*). By varying the length of the lead-alone segment, while holding that of the *lag-alone* segment constant, we would presumably be altering the latter proportionality (Fig. 4D). If the scenario above were true, then the ratio of lead to lag-directed saccades would likewise scale as the length of the lead-alone segment was varied (Fig. 6D). This was not observed. Our observations suggest that the owl, instead, treats the neural images of the lead and lag sound sources independently.

If delay *per se* does not cause localization dominance, what causes the leading sound to dominate perception when the delay is short? One possibility is that neurons responding to the leading sound preempt the responses of those that are selective for the location of the lagging sound by a lateral-inhibition-like process [11], [38].

Examination of the response dynamics of space map neurons, however, provides a simpler explanation. As shown in the PSTH of Figure 2A, space map neurons typically respond to the presentation of a sound from a single source with an initial burst of activity that decays rapidly over the course of the sound to a near steady-state. For paired lead/lag stimuli, the leading sound evokes the same vigorous onset response until the lagging sound is activated (Fig. 2D), at which point the response decreases to that which is evoked by two uncorrelated noise bursts (Fig. 2C). When the leading sound ends, neurons representing the lag location may begin to fire (Fig. 2E) but the response is less vigorous and roughly equivalent to that which occurs near the end of the single-source PSTH (Fig. 2A).

**Figure 7:**
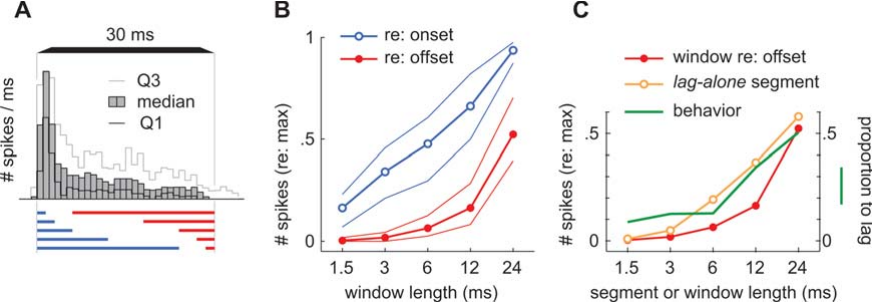
Potential explanation for localization dominance in the responses of neurons to single sounds. (A) PSTH showing the median neural responses that were evoked, in our sample of cells, by single-source *targets* (shown also in Fig. 2A). (B) Neural responses measured in time-windows of length and position that were the same as the *lead-alone* (onset; blue lines) or *lag-alone* segments (offset; red lines) under the standard paradigm. (C) Median responses evoked by *lag-alone* segments (orange line; shown also in Fig. 3A) and by single sounds during equivalent time-windows (red line; redrawn from panel A). The green line shows the proportion of lag-directed saccades for all subjects under the standard paradigm (shown also in Fig. 6A).

That localization dominance might be explained by the response dynamics of neurons to single sounds is shown in Figure 7. Figure 7A shows the PSTH that was derived from the responses of all neurons in our sample to a single sound source (shown also in Figure 2A). Figure 7B shows the cumulative response that was evoked during a variable time-window that encompassed either the onset or the offset of the single source (as indicated by lines below the PSTH of Fig. 7A). As expected, the cumulative response was far weaker for time-windows that encompassed the sound's offset (red lines). Figure 7C compares these responses, at the sound's offset (red line, redrawn from Fig. 7B), with those that were evoked during lag-alone segments of equivalent length (orange line; shown also in Fig. 3A). These responses are directly comparable because most of the response to the lagging sound was evoked during the lag-alone segment. Finally, the green line in Figure 7C shows the proportion of lag-directed saccades for all subjects in our standard paradigm (shown also in Fig. 6A). Taken together, these data suggest that the owl may rarely saccade to the source of the lagging sound, when the delay is short, because the lag-alone segment emerges from the superposed segment only shortly before it ends. As delay increases, the lag-alone segment begins to evoke a neural response, at the lead's offset, that closely resembles the steady-state portion of the neural response to a single-source. Viewed in this way, the weaker representation of the lag source need not be attributed to a lateral inhibition-like process [11], [38], although such a process is not excluded.

Localization dominance, as its name implies, has to do with spatial information. It is well known that even at delays where localization dominance is thought to operate, human listeners are aware of a reflection although they cannot localize it. Indeed, circuitry has been proposed that moderates the inhibition of responses to the lagging source to explain this observation [39], [40]. If the lag-alone segment is crucial for the localization of the lag source, did we render the owl “unaware” of the lagging source when we removed the lag-alone segment? The physiological data suggests not. As shown in Figure 3B, the lag source does, in fact, still evoke a response during the superposed segment, and this response may provide the basis for an “awareness” of a reflection, without the need for a specialized circuit. In this context, the lag-alone segment may be critical for *precise* localization but not for “awareness” of a reflection.

It is of interest to determine whether our results in owls generalize to human listeners. Although there are a number of studies of the precedence effect that have used stimuli that are long enough to overlap when the onset-delay is short [34], [37], [41]–[43], we are not aware of any that replicate our study in owls. An analogy is found, however, in a study by Perrott and Baars [44] to determine the relative contributions of the transient time differences at the onsets and offsets of binaurally-presented stimuli and ongoing, interaural, time differences. In one of their experimental conditions, human listeners heard binaurally correlated noise bursts in which the signal in one ear was terminated before that in the other. There was thus a segment of time when the signal was present in both ears (i.e., a binaurally superposed segment) followed by a monaural lag-alone segment, the length of which was varied by the experimenters. As the offset time was varied, the subjects were asked to identify the ear in which the signal terminated earlier. Perrott and Baars [44] reported anecdotally, that when the monaural lag-alone segment was at least 2–3 ms, subjects experienced both a centrally located (fused) image and a separate sound in the ear with the monaural lag-alone segment. As suggested by our data for the owl, the additional sound was heard as a separate auditory event when its waveform had emerged sufficiently from the superposed segment.

Finally, our results may appear, at first glance, to conflict with studies showing that humans experience localization dominance even when the lead-alone and lag-alone segments of stimuli were removed [34], [43]. Our results may not contradict these findings because their stimuli, unlike ours, were narrow-band or low-pass filtered and were more deeply amplitude-modulated as a result. Had we presented stimuli with comparable amplitude modulations, leading targets might have evoked increased firing throughout the superposed portions of our stimuli as delay was increased. This is because each transient peak in the leading sound's envelope is likely to form a brief lead-alone segment while each peak in the lagging sound's envelope is likely to form a brief lag-alone segment, superposed segment, or both. Preliminary results suggest that the envelopes of our stimuli may have been too shallow to form these presumptive lead- and lag-alone segments during the superposed portions of our stimuli [45]. Echo-threshold, in our experiments, thus appears to be related to the only available *lag-alone* segment and the neural responses on the space map that were elicited by it.

## Materials and Methods

### Animals

Experiments were carried out in 6 captive-bred adult barn owls, Tyto alba, under a protocol approved by the Institutional Animal Care and Use Committee of the University of Oregon. The birds were held in our colony under a permit from the US Fish and Wildlife Service.

### Single unit recordings

Neurophysiological experiments were conducted in a single-walled sound-attenuating chamber (Industrial Acoustics Co.; 1.8 m^3^) equipped with a stereotaxic frame and surgical microscope.

Single unit recordings were obtained from the space maps of 3 birds anesthetized with a mixture of ketamine and Diazepam. Anesthesia was induced by an intramuscular injection of ketamine (22 mg/kg) and Diazepam (5.6 mg/kg) and maintained with additional doses as needed (typically, every 2–3 hrs after induction).

Upon induction of anesthesia, the owl was given an injection of physiological saline (10 cc; subcutaneous). Electrocardiographic leads and an axial temperature probe were attached and the owl was wrapped in a water-circulating blanket and placed in a stereotaxic frame. Axial body temperature was maintained between 35° and 40° C. Its head was secured to the frame by a previously implanted head-post. Earphones (Etymotic Research, ER-1) were inserted into the ear canals, and a recording-well, implanted previously [46], was opened and cleaned to admit a glass-coated tungsten electrode (1 MΩ<impedance<12 MΩ at 1 kHz). The electrode's output was amplified and then fed to a signal processor (Tucker-Davis Technologies, RP2), oscilloscope, and audio monitor.

The electrode was advanced by a stepping motor microdrive (D-500, Power Technologies) while search stimuli, consisting of 100 ms bursts of broadband noise (2–11 kHz), were presented over the earphones in virtual auditory space (VAS; see below). A custom-built interactive graphical user interface (Matlab, Mathworks) was used to control the stimuli. The space map of the ICx was typically found 14–17 mm deep to the telencephalic dural surface. The activity of a single neuron was isolated using level detection and the time of each spike was recorded. Upon isolation, the unit's SRF was charted by presenting bursts of broadband noise (2–11 kHz; 100 ms) from each of 290 loci in the frontal hemisphere. The neurons responded vigorously to the broadband stimuli and had well-restricted SRFs with sizes and shapes consistent with those described in earlier studies using stimuli in the free-field [18], VAS [21], or both [29].

Typical recording sessions involved 1 to 3 electrode penetrations and lasted less than 12 hrs. At the end of a session, the well was rinsed with a 0.25% solution of chlorhexidine and sealed. The owl was placed in an isolated recovery chamber until it recovered from anesthesia, at which point, it was returned to its flight enclosure.

### Neurophysiological stimulus presentation

For neurophysiology, stimuli were presented in virtual auditory space (VAS) generated using *individualized* head related transfer functions [HRTF, 29]. For each bird, the HRTF for each ear was measured from 613 locations in the frontal hemifield at a resolution of 5° in azimuth and elevation (Fig. 1F). HRTFs were band-pass filtered between 2 and 12 kHz and stored digitally as 255-point (8.5-ms; 30-kHz) finite impulse response filters (HRIR). The stimuli, which consisted of variable duration noise bursts (Fig. 1A–D; 2–11 kHz; 2.5 ms linear ramps), were convolved with the HRIRs, converted to analog at 48.8-kHz, amplified (RP2.1, HB6, Tucker-Davis Technologies), and presented over earphones. Each stimulus condition was repeated 50–100 times with a 350 ms inter-stimulus interval. Stimuli were presented 30 dB above the response threshold of each space-specific neuron at its best location.

We characterized each isolated unit's SRF by presenting sounds from 290 virtual locations. The “best location” within each SRF was defined as the location that evoked the maximum average number of spikes across each repetition. Stimuli that were presented from this location in subsequent tests are referred to as *targets*. The stimulus placed outside the SRF is referred to as the *masker*. The location of the *masker* was always diametrically opposed to that of the *target* in polar coordinates (Fig. 1F).

In trials to probe the precedence effect, broadband noise-bursts were presented with various lead- and lag-alone segment lengths from the *target* and *masker* locations. The noise bursts were identical (i.e., 100% correlated) except for the delay. Before filtering with the HRTF, the noises were flat (<±1 dB) between 2–11 kHz and had trapezoidal envelopes (2.5 ms rise and fall times). For each cell, we also presented a pair of *independent* (i.e., 0% correlated) noise bursts simultaneously from *both* the *masker* and *target* loci, as well as noise-bursts from a single source at the location of the *masker* or the *target*.

### Neurophysiological data analysis

Spike times were adjusted to account for each single-unit's response latency (11 ms, 2.75 ms; median, inter-quartile range). Peri-stimulus time histograms (PSTH; 1-ms bins) were then produced for each stimulus configuration. Each cell's response was normalized using the maximum number of spikes evoked, within a single bin (usually the first or second bin after 0-ms), by a single source emitting 30-ms noise-bursts presented from the center of each cell's SRF [11]. In addition, we analyzed each response by counting the overall number of spikes that each *target* evoked during its entire duration or during its *lead-alone*, *lag-alone*, or *superposed* segment. These responses were normalized to the response evoked, in each cell, by single 30-ms sounds presented from the center of its SRF [11]. We used non-parametric statistics because variation was often skewed in a positive direction when responses were weak.

### Behavioral subjects

Three barn owls (*C*, *S*, and *T*) housed together in a single enclosure within our breeding colony were hand-reared and trained to make head saccades toward single visual and auditory stimuli for a food reward. The owls' weights were maintained at ∼90% of the free-feeding level during training and testing.

### Behavioral apparatus

Behavioral experiments were conducted in a double-walled anechoic chamber (Industrial Acoustics Co. IAC; 4.5 m×3.9 m×2.7 m). An owl was tethered to a 10×4 cm perch mounted atop a 1.15 m post in the chamber. A dispenser for food-rewards was also attached to the post near the owl's talons. A custom-built, head-mounted, magnetic search-coil system (Remmel Labs) was used to measure the owl's head movements in azimuth and elevation [28], [47]. The coils were calibrated before each session, to ensure accuracy within ±1° after adjusting for small differences in the angles with which head-posts were attached to each owl's skull.

Sounds were presented from 10 dome tweeters (2.9-cm, Morel MDT-39), each attached to the end of a ∼1±0.5 m flexible arm (Loc-Line). Speaker distance was 1.5 m. Speaker pairs were positioned with opposite polar angles (Fig. 5) in reference to the location of a central fixation LED (2.9 mm, λ = 568 nm). Radii (r) were standardized across speaker pairs at 10°, 15°, 20°, 25°, and 30°. New polar angles were generated randomly for each speaker pair every 2 to 4 test sessions. Subjects were monitored continuously throughout test sessions using an infrared camera and infrared light source (Canon Ci-20R and IR-20W, Lake Success, NY).

### Behavioral stimuli

Stimuli were the same as those used in our physiological experiments except that they were presented in the free-field rather than in VAS. Trials in a session consisted primarily of single-source trials (∼80%), amongst which we randomly interspersed paired-source trials with stimuli having lead- and lag-alone segments of various lengths. In the single-source trials, stimulus durations were roved between values that corresponded to the total lengths of the paired stimuli. Stimuli were converted to analog at 48.8-kHz and routed to speakers using two power multiplexers (RP2.1, HB7, PM2R, Tucker-Davis Technologies). Stimulus sound-pressure level (SPL) was roved in 1 dB increments between 27 and 33 dB across trials, in reference to the output of an acoustic calibrator (re: 20 µPa, 4321, Brüel & Kjær). Stimuli were presented with equal SPL on paired-source trials. Amplitude spectra were flat (±1.5 dB, 2–11 kHz) when measured with a microphone (4189, Brüel & Kjær) positioned at the location of the owl's head.

### Behavioral paradigm

During the training phase, owls were rewarded for making head saccades to the centering LED. Once subjects began making these saccades, they were rewarded for turning to acoustic stimuli (6 to 54 ms noise –bursts) after fixating on the centrally located LED.

Test sessions began once subjects learned to initiate trials by fixating the LED. Test sessions were included in our analyses once subjects began making saccades to within 5–8° of a single source. Each session consisted of 20 to 80 trials. A single noise stimulus of variable duration (6, 12, 24, 30, 42, 54 ms) was presented in approximately 80% of trials from a randomly selected speaker location. Randomly interspersed with these single-source trials were paired-source trials in which stimuli, described above, were presented from diametrically opposed speakers.

Head orientation and angular velocity were monitored continuously (20 Hz, RP2, Tucker-Davis Technologies) throughout each test session. Stimuli were presented only after the owl fixated on the centrally located LED for a random period of from 0.5 to 1 s. The LED was extinguished as soon as subjects oriented to within 3° of the LED. Trials were aborted if the owl moved beyond this 3° radius or if head velocity exceeded 2.6°/s before stimuli were presented. Once a trial was initiated, measurements were sampled at a rate of 1 kHz for 4.5 s, starting 1 s before the stimulus' onset. Subjects were automatically fed a small piece of a mouse (∼1 g), after a ∼1 to 3 s delay, if saccade velocity decreased to less than of 4°/s and if the saccade ended at a location that was within 5–8° from a previously activated speaker or member of a speaker pair.

### Behavioral data analysis

After measuring baseline head-velocity for 250 ms prior to stimulus presentation, the beginning of each saccade was determined as the time at which velocity exceeded this baseline measure plus 5 s.d. for 50 ms. Saccade latency was measured as the time from the onset of each stimulus to the beginning the saccade that was evoked by the stimulus. The end of each saccade was determined as the time at which velocity decreased to below baseline plus 8 s.d. for 50 ms. Saccades were subject to considerable analysis. Here, however, we were concerned only with the speaker toward which a saccade was directed during a paired-source trial. Because subjects were first required to fixate on a centrally located LED, and because each speaker-pair was separated by ∼180°, it was easy to determine whether saccades were lead- or lag-directed. Localization dominance was therefore estimated simply as the proportion of trials on which saccades were lag-directed. Saccade angles were rarely >75° (absolute polar coordinates re: either source at 0°; see Fig 5; <0.5–1% of trials/subject) and were never equal with respect to each paired-source (±90°). In contrast with a similar previous study [28], saccades were rarely directed first toward one speaker and then toward the second, after making a ∼180°, hairpin, turn (<0.5% of trials/subject). Reversals in saccade direction that did occur were as likely to occur on single-source trials and almost always corresponded with a spurious head movement prior to stimulus presentation.

## Supporting Information

Figure S1 Raster plots and peri-stimulus time histograms (PSTH) showing the spikes evoked in a single unit by a subset of stimuli (unit: 1027LF215, 50 repetitions, 1-ms PSTH bins). (A) Responses evoked by leading targets under the standard paradigm when lead and lag sounds were of equal length. (B) Responses evoked by lagging targets under the standard paradigm. (C) Responses evoked by lagging targets when the length of the lag-alone segment was experimentally increased to 12 ms (1.5, 3 and 6 ms delays) or decreased to 6 ms (12 and 24 ms delays). (D) Responses evoked by single targets presented from the center of the cell's spatial receptive field (10° azimuth, 25° elevation). Each PSTH was normalized using the maximum number of spikes evoked by this single target (D).
(0.31 MB PDF)

Figure S2 Saccade latency (A) and localization error (B) measured separately for each bird (C, S, and T). Plotted are the median values for lead-directed (filled symbols) and lag-directed saccades (open symbols) and for saccades directed towards single sound sources (black line). For paired stimuli (colored lines), the abscissa shows only the length of each stimulus' lag-alone segment. The stimuli were thus grouped without respect to the length of the lead-alone segment and responses to stimuli with lag-alone segments < = 3 ms were combined. Values for single sound sources (black lines) were measured when these sounds had durations equal to those of the paired stimuli (i.e., 3 = 30, 6 = 36, 12 = 42, 24 = 54 ms). Error bars show the first and third quartiles. Values without error bars are the averages of 2 data points and should be viewed with caution. (A) Saccade latency. Latencies were consistently greater when saccades were directed towards paired stimuli (colored lines), in comparison with when saccades were directed towards single sound sources (black lines; P<110−6; df = 11; Kruskal-Wallis; Dunn-Holland-Wolfe multiple comparisons). Latency increased further when saccades were lag-directed and when the length of the lag-alone segment was short (<12 ms). These trends should be viewed with caution, however, since saccades were rarely lag-directed when the lag-alone segment was short (Fig. 6). Latency did not differ significantly when stimuli were lead-directed, even when the length of the lag-alone segment was decreased to < = 3 ms (including latencies for subject S). (B) Localization error. Error, ε, was measured, in Cartesian coordinates, as the angular distance from where each saccade ended to the nearest speaker (ε = [ε azimuth2+ε elevation2]1/2 ). Saccades were nearly as accurate and precise as those to single sound sources, except for when the saccades were lag-directed and when the lag-alone segment was short (<12 ms). These trends should be viewed with caution, however, since saccades were rarely lag-directed when the lag-alone segment was short (Fig. 6).
(0.49 MB PDF)
