# Supplementary material for: Independence of Echo-Threshold and Echo-Delay in the Barn Owl
Source: PLoS One. 2008 Oct 31;3(10):e3598. doi: 10.1371/journal.pone.0003598 (PMC2571984; doi:10.1371/journal.pone.0003598)
Supplement: Figure S1 — Raster plots and peri-stimulus time histograms (PSTH) showing the spikes evoked in a single unit by a subset of stimuli (unit: 1027LF215, 50 repetitions, 1-ms PSTH bins). (A) Responses evoked by leading targets under the standard paradigm when lead and lag sounds were of equal length. (B) Responses evoked by lagging targets under the standard paradigm. (C) Responses evoked by lagging targets when the length of the lag-alone segment was experimentally increased to 12 ms (1.5, 3 and 6 ms delays) or decreased to 6 ms (12 and 24 ms delays). (D) Responses evoked by single targets presented from the center of the cell's spatial receptive field (10° azimuth, 25° elevation). Each PSTH was normalized using the maximum number of spikes evoked by this single target (D). (0.31 MB PDF) [file pone.0003598.s001.pdf]

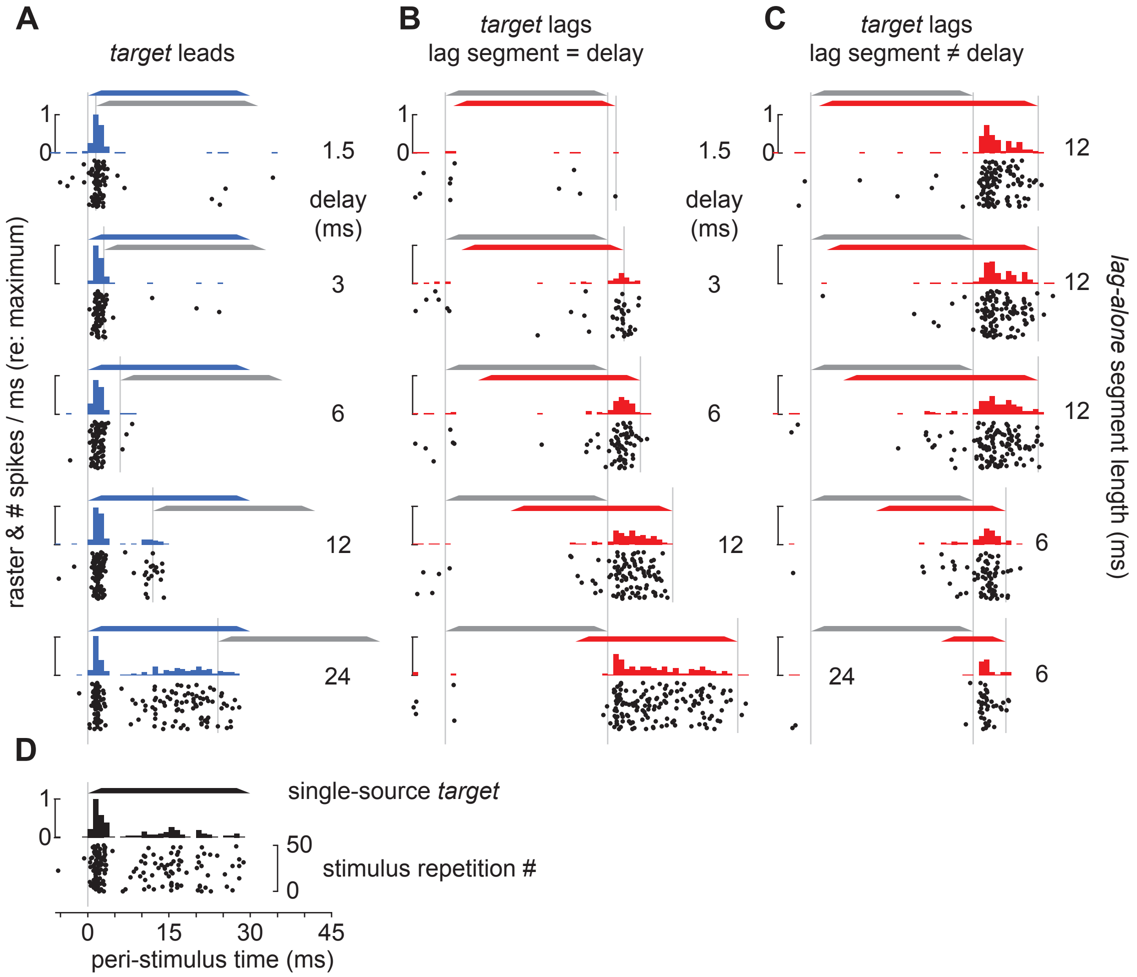

Supplementary Figure 1 Raster plots and peri-stimulus time histograms (PSTH) showing the spikes evoked in a single unit by a subset of stimuli (unit: 1027LF215, 50 repetitions, 1-ms PSTH bins). (**A**) Responses evoked by leading *targets* under the standard paradigm when lead and lag sounds were of equal length. (**B**) Responses evoked by lagging *targets* under the standard paradigm. (**C**) Responses evoked by lagging *targets* when the length of the *lag-alone* segment was experimentally increased to 12 ms (1.5, 3 and 6 ms delays) or decreased to 6 ms (12 and 24 ms delays). (**D**) Responses evoked by single *targets* presented from the center of the cell's spatial receptive field (10° azimuth, 25° elevation). Each PSTH was normalized using the maximum number of spikes evoked by this single *target* (**D**).
