# Supplementary material for: Independence of Echo-Threshold and Echo-Delay in the Barn Owl
Source: PLoS One. 2008 Oct 31;3(10):e3598. doi: 10.1371/journal.pone.0003598 (PMC2571984; doi:10.1371/journal.pone.0003598)
Supplement: Figure S2 — Saccade latency (A) and localization error (B) measured separately for each bird (C, S, and T). Plotted are the median values for lead-directed (filled symbols) and lag-directed saccades (open symbols) and for saccades directed towards single sound sources (black line). For paired stimuli (colored lines), the abscissa shows only the length of each stimulus' lag-alone segment. The stimuli were thus grouped without respect to the length of the lead-alone segment and responses to stimuli with lag-alone segments < = 3 ms were combined. Values for single sound sources (black lines) were measured when these sounds had durations equal to those of the paired stimuli (i.e., 3 = 30, 6 = 36, 12 = 42, 24 = 54 ms). Error bars show the first and third quartiles. Values without error bars are the averages of 2 data points and should be viewed with caution. (A) Saccade latency. Latencies were consistently greater when saccades were directed towards paired stimuli (colored lines), in comparison with when saccades were directed towards single sound sources (black lines; P<110−6; df = 11; Kruskal-Wallis; Dunn-Holland-Wolfe multiple comparisons). Latency increased further when saccades were lag-directed and when the length of the lag-alone segment was short (<12 ms). These trends should be viewed with caution, however, since saccades were rarely lag-directed when the lag-alone segment was short (Fig. 6). Latency did not differ significantly when stimuli were lead-directed, even when the length of the lag-alone segment was decreased to < = 3 ms (including latencies for subject S). (B) Localization error. Error, ε, was measured, in Cartesian coordinates, as the angular distance from where each saccade ended to the nearest speaker (ε = [ε azimuth2+ε elevation2]1/2 ). Saccades were nearly as accurate and precise as those to single sound sources, except for when the saccades were lag-directed and when the lag-alone segment was short (<12 ms). These trends should be viewed with caution, howe [file pone.0003598.s002.pdf]

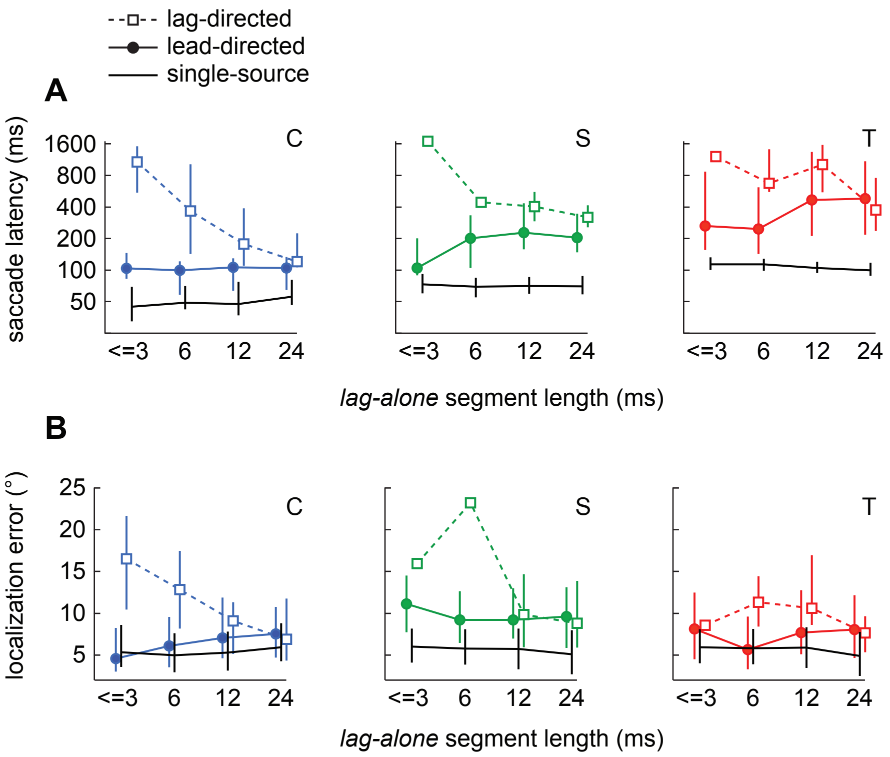


**Supplementary Figure S2** Saccade latency (**A**) and localization error (**B**) measured separately for each bird (C, S, and T). Plotted are the median values for lead-directed (filled symbols) and lag-directed saccades (open symbols) and for saccades directed towards single sound sources (black line). For paired stimuli (colored lines), the abscissa shows only the length of each stimulus' lag-alone segment. The stimuli were thus grouped without respect to the length of the lead-alone segment and responses to stimuli with lag-alone segments <= 3 ms were combined. Values for single sound sources (black lines) were measured when these sounds had durations equal to those of the paired stimuli (i.e., 3=30, 6=36, 12=42, 24=54 ms). Error bars show the first and third quartiles. Values without error bars are the averages of 2 data points and should be viewed with caution. (**A**) Saccade latency. Latencies were consistently greater when saccades were directed towards paired stimuli (colored lines), in comparison with when saccades were directed towards single sound sources (black lines; P<110-6; df=11; Kruskal-Wallis; Dunn-Holland-Wolfe multiple comparisons). Latency increased further when saccades were lag-directed and when the length of the lag-alone segment was short (< 12 ms). These trends should be viewed with caution, however, since saccades were rarely lag-directed when the lag-alone segment was short (Fig. 6). Latency did not differ significantly when stimuli were lead-directed, even when the length of the lag-alone segment was decreased to <= 3 ms (including latencies for subject S). (**B**) Localization error. Error, ε, was measured, in Cartesian coordinates, as the angular distance from where each saccade ended to the nearest speaker ($\varepsilon = [\varepsilon_{\text{azimuth}}^2 + \varepsilon_{\text{elevation}}^2]^{1/2}$). Saccades were nearly as accurate and precise as those to single sound sources, except for when the saccades were lag-directed and when the lag-alone segment was short (< 12 ms). These trends should be viewed with caution, however, since saccades were rarely lag-directed when the lag-alone segment was short (Fig. 6).
